# Aplasie cutanée congénitale du vertex chez le nouveau-né

**DOI:** 10.11604/pamj.2013.16.148.3056

**Published:** 2013-12-20

**Authors:** Rachid Abilkassem, Aomar Agadr

**Affiliations:** 1 Service de Pédiatrie, Hôpital Militaire d’Instruction Mohamed V, Université Med V, Souissi, Rabat, Maroc

**Keywords:** Aplasie cutanée congénitale du vertex, nouveau-né, Aplasia cutis congenita of the vertex, newborn

## Image en médicine

Nouveau-né de sexe masculin, né à terme par voie basse avec une bonne adaptation à la vie extra utérine, pesant 3500g. L’inspection à la naissance a montré une petite zone médiane au niveau du vertex, ulcérée, érythémateuse, mesurant 3 cm sur 2 cm, non hémorragique. Cette zone d’aplasie n’est pas associée à un défect osseux à la palpation. L’examen somatique notamment neurologique était sans particularité. L’exploration cérébrale (radiographie de crane, échographie transfontanellaire, scanner cérébral) était normale. La lésion a évolué vers la cicatrisation après des soins locaux biquotidiens au tulle gras. Il persiste une alopécie cicatricielle du vertex et une zone croûteuse centrale.

**Figure 1:**
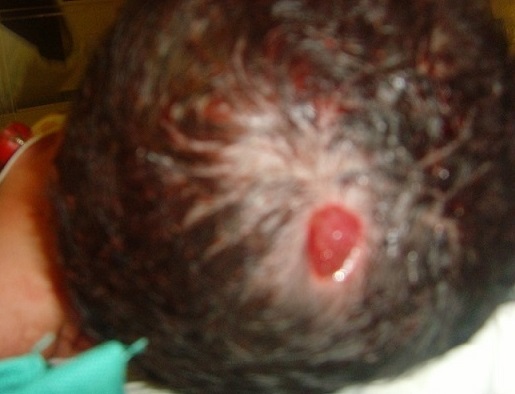
Petite zone médiane au niveau du vertex, ulcérée, érythémateuse mesurant 3cm sur 2cm

